# Gait Characteristics of Transtibial Amputees on Level Ground in a Cohort of 53 Amputees - Comparison of Kinetics and Kinematics With Non-amputees

**DOI:** 10.33137/cpoj.v2i2.32955

**Published:** 2020-01-15

**Authors:** E Pröbsting, M Bellmann, T Schmalz, A Hahn

**Affiliations:** 1 Clinical Research & Services / Biomechanics, Ottobock SE & Co. KGaA, Hermann-Rein-Straße 2a, 37075 Göttingen, Germany.; 2 Clinical Research & Services, Ottobock Healthcare Products GmbH, Brehmstraße 16, 1110 Vienna, Austria.

**Keywords:** Prosthesis, Transtibial Amputees, Gait Analysis, Kinematic, Kinetic, Amputation, Gait Velocity, Step Length, Gait Asymmetry

## Abstract

**STUDY DESIGN:**

Retrospective analysis

**BACKGROUND:**

The gait characteristics of transtibial amputees (TTs) have been described many times. In general, the literature reported nearly consistent results for the kinematic and kinetic parameters of the prosthetic side. However, the literature revealed inconsistent findings on kinetic parameters for determining the risk of developing knee osteoarthritis, such as the peak knee adduction moment, knee flexion moment and vertical ground reaction forces.

**OBJECTIVES:**

The objective of our study was to describe the sagittal kinetic and kinematic gait characteristics of the ankle and residual knee joint of the prosthetic limb and the knee loading parameters of the sound side of unilateral TTs. This specific consideration may contribute to resolving the controversy of these parameters in the literature.

**METHODS:**

We analysed our database containing gait analyses from 53 unilateral TTs and compared data to a control group (CG), also taken from our database. The sagittal kinetic and kinematic gait characteristics of the ankle and residual knee joint of the prosthetic limb, and selected knee loading parameters of the sound side (the peak knee adduction moment, knee flexion moment and vertical ground reaction forces) were evaluated. Beside these parameters we reported typical spatiotemporal gait parameters as gait velocity, step length, step length asymmetry, stance phase duration and asymmetry of stance phase duration.

**RESULTS:**

The TTs walked slower and more asymmetrically than the CG. The kinematic pattern of the prosthetic ankle differed from that found in the CG. The largest difference was observed for the range of motion of the plantarflexion at push-off, which was significantly reduced for the prosthetic foot. The residual knee joint was generally affected with respect to decreased moments and reduced knee flexion during stance phase. The peaks of the vertical ground reaction forces and knee adduction moments showed no differences between the sound side of amputees and the CG. The peak knee flexion moment at midstance was significantly reduced for the sound side of amputees in comparison with the CG.

**CONCLUSION:**

The biomechanical data measured for the prosthetic side in a cohort of 53 unilateral TT amputees conformed with the literature. The parameters determining the risk of developing knee osteoarthritis investigated in our retrospective analysis were not increased on the sound side in comparison with non-amputees. We deem it reasonable to assume that an appropriate prosthesis will reduce the likelihood of overloading the knee on the sound side during normal walking.

## INTRODUCTION

The gait characteristics of a transtibial amputee (TT) with a prosthesis significantly deviate from normal gait patterns.^1,2^ Literature primarily analysed the effect of components, weight distributions or sockets, typically for a small number of patients.^1,2^ In general, from all of these studies, it can be concluded that TT amputees walk with slower velocity,^3,4^ shorter steps^3,4^ and longer stance duration on the sound limb5 compared to non-amputees.

Beside these temporalspatial parameters the results of kinetic and kinematic parameters enable a more detailed evaluation for the gait of TTs. Research studies^3–18^ reporting such findings for TTs in comparison to nonamputees were summarized in Table 1.

**Table 1:**
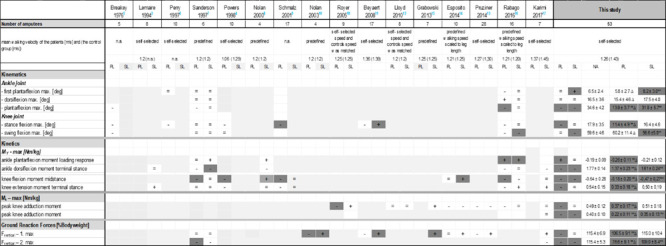
left: Comparison of the results of the TTs (transtibial amputees) walking with a conventional or ESR prosthetic foot, for the sound leg (SL) and the prosthesis leg (PL) with the control group of non-amputees (NA) in the individual studies. Significant differences in the individual study between TTs and controls are marked in bold and grey. In the studies of Breakey5 and Lemaire3, no statistical data were given. Please note that the data are extracted in different ways. Right: Results of the present study. Mean peak values of selected kinetic and kinematic parameters including information about statistical comparisons between TT and control group of NA and about statistical comparison between prosthetic and sound limb. “=”: difference between values of TTs and CG <= 2 deg, 0.05 Nm/kg or 2% BW; “+”: value is increased for TTs; “-“: value is reduced for TTs; “*”: p≤0.05 for comparison between TTs and CG; “**”: p≤0.01 for comparison between TTs and CG; "∆": p≤0.05 for comparison between SL and PL

Most kinematic analyses of the prosthetic side reported a reduced range of plantarflexion in late stance and early swing^5,7,16^ in TTs in comparison with non-amputees. Furthermore, knee flexion was altered on the prosthetic side. Knee flexion was reduced during stance phase^4,5,6,9,11,16^ and some studies showed a reduced peak knee flexion angle during swing.^5,16^ The kinematic pattern of the sound limb in TTs appeared to be comparable to that of non-amputees.^7^

Kinetic analyses of the prosthetic side consistently reported a lower external dorsiflexion moment in late stance with the prosthetic foot as compared to nonamputees.^3,7^ Furthermore, the knee joint on the affected side showed a markedly reduced external flexion moment during midstance.^4, 9^

However, literature revealed inconsistent results with respect to kinetic compensatory adaptations on the sound side, specifically the peak knee adduction moment, knee flexion moment and vertical ground reaction forces.

Reports on the knee adduction moments on the sound side were conflicting: Some studies showed no differences,^12,16,17^ others reported increases^13,18^ by trend, and a few studies^14,15^ found a reduction in comparison to controls. A similar controversy could be found for the comparison of the sound side knee flexion moments at midstance. A few studies showed a significant increase on the sound side in comparison to the controls^8,14^ others reported no differences^7,9,17^ or a decrease by trend.^16^

Both parameters knee flexion and adduction moment refer to knee loading parameters influencing the risk of developing knee osteoarthritis. The relevance of the external knee adduction moment regarding the development of knee joint degeneration in the general population^19^ was highlighted. Particularly, the first peak of the external knee adduction moment during stance has been associated with the severity of knee osteoarthritis.^20,21^ Furthermore, the peak external knee flexion moment during midstance was considered as another predictor of knee loading^22^ and the first peak of the vertical ground reaction force was also increased for patients with knee osteoarthritis.^20^ The latter also showed diverging results for the sound side of TTs in the literature: A significantly increased first peak on the sound side in comparison to healthy controls was reported in two studies.^10,13^ Some studies showed a statistically non-significant increase,^11,14,15,17^ while other studies showed no differences at all.^6, 7^

As the investigations cited did not show consistent results, the described higher prevalence of knee osteoarthritis on the sound side in TTs^21^ could not be explained with certainty to be caused by higher loads on this knee.

Different studies showed that the parameters on the sound side are mainly influenced by the prosthesis^13,14,23^ therefore the prosthetic side should also be analysed to evaluate compensatory adaptations on the sound side.

In the present study we retrospectively analysed the TT population from our database including a large sample size to generate a representative cross section of this cohort and compared it to able-bodied individuals. The objective of our study was to compare the gait characteristics regarding the sagittal kinetic and kinematic parameters of the ankle and knee joints of the prosthetic limb for a large sample of unilateral TTs. Additionally, we have also examined the knee loading parameters on the sound side as indicators for determining the risk of developing knee osteoarthritis. This specific consideration may contribute to resolving the controversy of these parameters in the literature.

## METHODOLOGY

### Data collection

Gait analyses with amputees have been performed in the Ottobock gait lab in Göttingen since 2002, a VICON system has been available and only data obtained from this system were analysed retrospectively for this study.

From 2002 to 2013, the experimental setup consisted of a 6 MX camera motion capture system (120 Hz; VICON, Oxford Metrics, UK) and from 2013 onwards of a 12-BONITA camera motion capture system (200 Hz; VICON, Oxford Metrics, Yarnton, UK). Two force plates (400 Hz; Kistler 9287A, Winterthur, CH) were positioned in the centre of the 12-metre walkway for measuring the bilateral ground reaction forces during one gait cycle.

Both systems were synchronised, they started simultaneously via a light triggered photo cell. Each subject conducted 8 to 15 single measurements of walking trials.

The database included 279 amputees of different amputation levels with 5594 different measurement situations. We identified one characteristic session for each TT with the following inclusion criteria:

-Unilaterally amputated

-Adults > 18 years

-No additional health impairment

-Walking with their self-selected velocity on level ground

-Prosthesis with a commercially available foot

-Prosthesis to be aligned according to the criteria defined by Blumentritt ^24^

Data from a control group (CG), which had been included for comparison purposes, were obtained from the same database. These adult individuals were screened for orthopaedic and neurologic impairments and were not limited by conditions that could have affected their gait. The CG consisted of 52 individuals (25 male/27 female). They were on average 32 (SD=12) years old, 1.75 (SD=0.10) m tall and weighed 72.6 (SD=12.2) kg.

All data analysed were collected at preferred self-selected and therefore comfortable and individually used walking speeds. The parameters of the groups were compared directly, even though the mean walking velocity differed between the amputees and the controls. The aim was to investigate the effect of normal, self-selected walking speed as an indication of the daily demands.

### Data analysis

Three-dimensional marker trajectories were tracked from 17 markers placed on anatomical landmarks (both sides: acromion, Epicondylus lateralis humeri, Processus styloideus ulnare, Trochanter major, compromise knee centre of rotation according to Nietert,^25^ Malleolus lateralis, Caput os metartasale IV and three asymmetric markers: left tibia, right thigh and left shoulder blade). This marker set has been used since 1998 and was created to analyse essential gait parameters for amputees. External joint moments were calculated based on ground reaction forces and coordinates of joint axes according to a previously described method.^26^

For the typical characteristics of the TT gait, the ankle and knee angle in the sagittal plane of both prosthetic and sound limbs and also the sagittal moments of these joints were evaluated.

Due to the different results in literature, the vertical ground reaction force, and the sagittal and frontal moments acting on the sound knee joint were evaluated in this study. The first peak of vertical ground reaction forces, the peak knee flexion moment during midstance and the first peak knee adduction moment were used for the statistical analysis as a possible key factor for developing osteoarthritis.

Moreover, spatiotemporal gait parameters were reported as well:

•Gait velocity

•Step length

•Step length asymmetry (the difference between both legs)

•Stance phase duration

•Stance phase duration asymmetry (the difference between both legs)

All kinetic and kinematic data were normalised to gait cycle (GC). The GC starts with the heel strike of one foot on the first force plate and ends with the following heel strike of the same side without touching the second force plate.

The peaks of the kinetic data used for the statistical analysis are defined in Table 1 and of the kinematic data are defined as follows:

-First plantarflexion max.: range of motion from ankle angle at heel strike to maximum of plantarflexion (at 5-20% GC)

-Dorsiflexion max.: range of motion from maximum of plantarflexion (at 5-20% GC) to maximum of dorsiflexion (at 40-60% GC)

-Plantarflexion max.: range of motion from maximum of dorsiflexion (at 40-60% GC) to maximum of plantarflexion (at 50-70% GC)

-Knee joint stance flexion: range of motion from knee angle at heel strike to maximum of knee flexion (at 10-30% GC)

-Knee joint swing flexion: range of motion from maximum of knee extension (at 30-50% GC) to maximum of knee flexion (at 50-70% GC)

Since all prosthetic feet used in this study have no ankle joints, terms like “dorsiflexion” and “plantarflexion” have to be handled with care in kinematic as well as in kinetic analyses. They were used to explain the deflection of the foot related to the natural motion.

### Statistical analysis

Mean values for all parameters were determined based on 8 to 12 gait cycles for the prosthetic and the sound limb. For the CG, the kinetic and kinematic data of the right leg and the spatiotemporal gait parameters were evaluated. Group means were calculated separately for each group based on the values of all TTs and the CG. Differences in peak values of biomechanical parameters between amputees and the CG were tested with the Mann-Whitney U-test, based on a non-normal distribution of all gait parameters tested with the Shapiro-Wilk test. The significance level was set at p < 0.05 for two-tailed tests. The peaks of the knee adduction moment, of the knee flexion moment during midstance and of the vertical ground reaction forces on the sound side are reported contradictorily in literature. Thus, their effect size (r=z/√N)) was calculated to assess the magnitude of the difference.^27^ As Cohen suggested, the effect size was defined with d=0.2 being considered a ‘small’ effect size, 0.3 represents a ‘medium’ effect size and 0.5 a ‘large’ effect size.^27^ If the effect size of the group comparison is 0.2 or smaller, the difference is marginal. Thus, the null hypothesis of the Mann-Whitney U-test stating that the two samples come from the same population and therefore show no differences is confirmed.

## RESULTS

### Individuals

The database contained data from 67 TTs, whereby 53 (39 male, 14 female) met the inclusion criteria.

The amputees were on average 48 (SD=16) years old, 1.77 (SD=0.09) m tall and weighed 84.3 (SD=17.8) kg. Twenty five individuals were amputated on the right and 28 on the left side. All individuals used passive prosthetic feet. The amputees’ mobility level (K-Level) was determined by subjective judgment of the prosthetist using the Medicare functional classification system (MFCL).^28^ Detailed information on the amputees is shown in Table 2.

**Table 2:** Transtibial amputees’ anthropometric data.

Patient	Height (cm)	Body mass with prosthesis (kg)	Age (yrs)	Follow up after amputation (yrs)	Reason for amputation	Gender (m/f)	Affected limb	K-level (1-4)	Prosthetic foot model
1	188	99	63	8	malignancy	m	right	2-3	C-Walk^1^
2	167	69	85	50	trauma	m	left	2	C-Walk^1^
3	181	113	48	23	trauma	m	right	3	Advantage DP^1^
4	182	72	22	8	trauma	m	left	3-4	C-Walk^1^
5	173	76	76	59	trauma	m	left	3	Dynamic Motion^1^
6	181	81	34	n.a.	trauma	f	left	3-4	C-Walk^1^
7	175	92	63	44	trauma	m	left	3	Dynamic Motion^1^
8	190	92	63	n.a.	arterial disease	m	left	2-3	C-Walk^1^
9	185	87	62	n.a.	n.a.	m	left	3	C-Walk^1^
10	182	79	41	9	trauma	m	left	4	Triton^1^
11	181	86	22	6	trauma	m	left	2	n.a.
12	172	87	66	2	arterial disease	m	left	1-2	Dynamic Foot^1^
13	160	61	46	21	trauma	f	left	2	Multiflex^2^
14	180	85	52	29	trauma	m	left	4	Triton^1^
15	193	115	43	15	trauma	m	left	4	Trias^1^
16	175	94	62	1	arterial disease	m	right	3	Dynamic Motion^1^
17	176	75	63	39	trauma	m	right	3	C-Walk^1^
18	171	69	22	3	trauma	f	right	3	C-Walk^1^
19	179	71	68	1	arterial disease	m	right	2	Dynamic Foot^1^
20	179	80	47	0	trauma	m	left	3	C-Walk^1^
21	187	88	71	8	trauma	m	left	3	C-Walk^1^
22	172	60	27	3	trauma	f	left	2-3	C-Walk^1^
23	170	70	34	6	arterial disease	f	left	3	Dynamic Motion^1^
24	156	63	44	1	arterial disease	f	right	2	SACH^1^
25	179	95	64	3	arterial disease	m	left	2-3	Dynamic Foot^1^
26	176	67	28	3	trauma	m	left	3	Dynamic Foot^1^
27	159	64	46	24	trauma	f	left	3	C-Walk^1^
28	186	79	29	29	congenital	m	left	4	C-Walk^1^
29	174	92	62	3	arterial disease	m	right	2	Dynamic Foot^1^
30	173	91	59	4	arterial disease	m	right	1-2	Greissinger Plus^1^
31	176	73	43	26	trauma	m	left	3-4	Dynamic Motion^1^
32	175	80,5	73	54	trauma	m	right	3	Dynamic Motion^1^
33	181	84	52	20	trauma	f	right	3	C-Walk^1^
34	180	92	65	4	arterial disease	m	left	3	C-Walk^1^
35	169	76,5	70	71	trauma	m	left	3	SACH^1^
36	203	144	25	8	trauma	m	right	2-3	Ceterus^3^
37	173	77	50	31	trauma	m	right	4	Triton^1^
38	176	93,5	53	1	sepsis	m	right	3-4	C-Walk^1^
39	178	117	28	1	trauma	m	right	3	Trias^1^
40	189	126	39	31	trauma	m	left	3	Axtion^1^
41	168	76	37	24	malignancy	f	right	3	Triton^1^
42	175	88	51	3	trauma	m	right	3	C-Walk^1^
43	174	75	43	n.a.	n.a.	f	left	2	n.a.
44	176	78	44	15	trauma	f	left	4	Triton^1^
45	183	113	26	2	malignancy	m	right	4	Triton^1^
46	165	69	47	2	arterial disease	f	right	3	Ceterus LP^3^
47	172	72	41	20	trauma	f	left	4	C-Walk^1^
48	156	53	40	33	trauma	f	right	3	Axtion^1^
49	177	79	47	10	arterial disease	m	right	4	Triton^1^
50	188	109	50	8	trauma	m	right	3-4	n.a.
51	168	70	28	20	trauma	m	right	4	Advantage DP^1^
52	187	102	33	17	trauma	m	right	4	Advantage DP^1^
53	183	69	48	2	infection	m	right	3	Trias^1^
**mean**	**176.9**	**84.3**	**48.0**	**16.7**		39 m	25 right		
SD	9.1	17.8	15.7	17.3		14 f	28 left		

Abbreviations: 1 Otto bock, Duderstadt D. 2 Endolite Blatchford, Hampshire, UK.

### Spatiotemporal gait parameters

TTs walked significantly slower than the CG (1.26 m/s vs. 1.43 m/s; p<0.001). Step length was significantly reduced: prosthetic side (0.71 m vs. CG: 0.76 m; p<0.05) and sound side (0.67 m vs. CG: 0.76 m; p<0.001). Step length asymmetry was significantly higher for the amputees (0.046 m; p<0.001) than for the CG (0.01 m). Stance phase duration on the sound side (65% of the gait cycle, GC) was significantly longer than on the prosthetic side (62% GC; p<0.001) and in the CG (61% GC; p<0.001). The asymmetry of the stance phase duration of 3% GC was significantly (p<0.001) increased.

### Foot/ankle kinematics and kinetics prosthetic side

**Figure 1:**
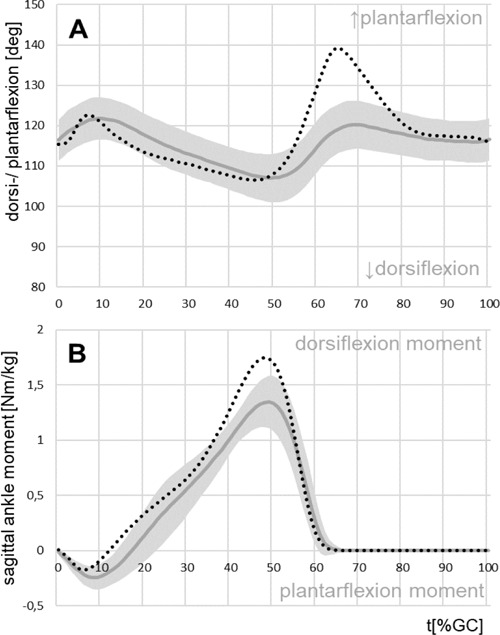
Mean parameters for the prosthetic side foot/ankle of the TTs with standard deviation (grey) and for the control group (dotted, black). A: mean pattern of foot/ankle motion, B: external sagittal moment acting on the ankle joint.

The kinematic pattern (Figure1A) of the prosthetic ankle differed from that found in the CG. The range of motion of plantarflexion in early stance on the prosthetic side (5.8°) was comparable to that of the CG (6.5°, p=0.2), while the movement was slower on the prosthetic side. The range of motion of the following dorsiflexion was also similar between both groups (15.4° vs. CG 16.5°; p=0.35). In the CG the movement was initially fast (up to 15% GC) and then it slowed down; between 10 and 50% GC, amputees’ dorsiflexion movement showed a constant velocity. The range of plantarflexion at the end of stance was significantly reduced on the prosthetic side (P: 13.9° and CG: 34.6°, Table 1). Additionally, there was negligible dorsiflexion during the swing phase of the prosthetic foot.

The maximum initial plantarflexion moment (Figure 1B) was significantly increased on the prosthetic side (-0.26 Nm/kg and CG: -0.19 Nm/kg; p=0.001). Peak dorsiflexion moment was significantly reduced for the prosthetic side (P: 1.34 Nm/kg and CG: 1.77 Nm/kg; p<0.001).

### Knee kinematics and kinetics prosthetic side

**Figure 2:**
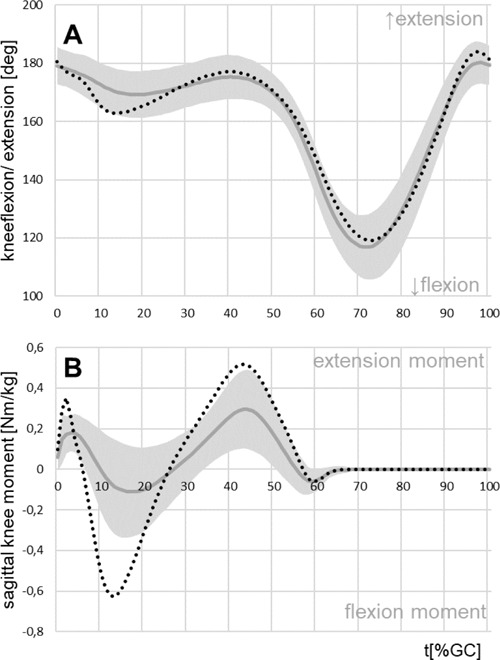
Mean parameters for the residual knee joint of the TTs with standard deviation (grey) and for the control group (dotted, black). **A**: mean pattern of knee motion, **B**: external sagittal moment acting on the knee joint.

For 46 out of 53 TTs, the prosthetic side showed a knee flexion during stance phase. The remaining seven TTs walked with fully extended knee. This motion ranged between 4 and 26 degrees with a mean stance phase flexion of 11.4° (Figure 2A, Table 1). This was significantly lower than in the CG (17.9°, p<0.001).

The range of motion during swing on the patients’ prosthetic side (60.2°) was similar to that of the CG (59.6°). The moment acting on the knee joint in the sagittal plane was generally reduced on the prosthetic side (Figure 2B).

### Sound side knee loading

**Figure 3:**
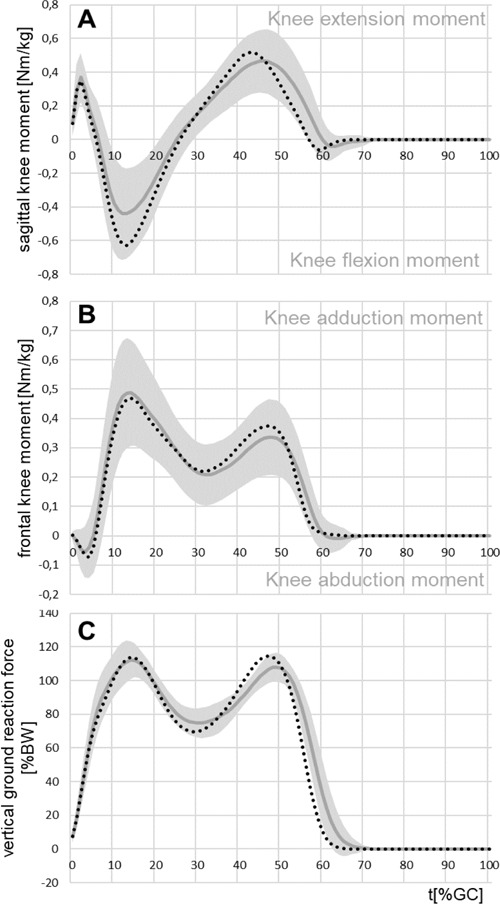
Selected mean parameters for the sound side of the TTs with standard deviation (grey) and for the control group (dotted, black). **A**: external sagittal moment acting on the knee joint, **B**: external frontal moment acting on the knee joint, **C**: vertical ground reaction force.

The peak values of the external knee extension moment showed no significant differences between TTs and CG. However, the peak external knee flexion moment was significantly reduced on the sound side (0.47 Nm/kg and CG: -0.64 Nm/kg; p<0.001; r=0.3) (Figure 3A and Table 1).

The first peak of the knee adduction moment showed no difference between the TTs (0.51 Nm/kg) and the CG (0.49 Nm/kg; p=0.747; r=0.03). Only the second peak was significantly reduced for the amputees (p=0.008) (Figure 3B). The mean first peak of vertical ground reaction force showed no significant difference between the sound side of amputees (115.0 %BW) and the CG (115.4 %BW, p=0.686). Only the mean second peak was significantly reduced for the amputees (p<0.001; r=0.04, Figure 3C).

## DISCUSSION

The objective of this study was to describe the gait characteristics with respect to the sagittal kinetic and kinematic parameters of the ankle and knee joints of the prosthetic limb of unilateral TTs. In view of the discrepancies of the sound side knee loading parameters found in the literature, these parameters were analysed in a representative large cross section of TTs (n=53) as well.

In the present study the TTs walked with a self-selected speed comparable with velocities cited in other studies (see Table 1), but significantly slower than the CG. The asymmetrical stance phase duration was also in accordance with the literature and was explained as “being the result of an early toe-off by the amputated limb owing to loss of the push-off function of ankle plantar-flexion”.^5^ The most obvious differences between TTs and the CG were seen at the prosthetic ankle, as previously described by Sanderson et al.^7^ With the TTs, the external plantarflexion moment in early stance was clearly increased and acts for a longer period. Also, plantarflexion was slower. This is in accordance with results of Breakey, who argued that the compression of the prosthetic heel could be responsible for the longer period between heel-contact and foot-flat in the amputated limb^5^ as well as the missing ankle joint. The constant velocity of the subsequent dorsiflexion and the steady increase of the corresponding moment are results of the lack of muscular control, especially of the triceps surae. Due to the removed plantar flexor muscles, the final plantarflexion at the end of stance was markedly reduced. As a result of this missing movement the peak dorsiflexion moment was subsequently reduced.^7^ The missing muscles controlling the ankle also affect the swing phase, because the prosthetic foot showed only a slight dorsiflexion, which could be due to elastic recoil after compression. In addition to the changes at the ankle, the residual knee of the prosthetic limb was systematically affected.

During stance, the range of motion of the residual knee of the prosthetic limb was significantly reduced, although TTs rarely suffer from pathological flexion or extension deficits of these joints. A long-term adaptation to the amputation is the reduction in strength^29,30^ and cross-sectional area^9,31^ of the muscles crossing the proximal joints. A possible consequence could be a reduction of the joint moments, which have to be compensated by the muscles.^9^ This reduction was also seen at the knee joint in this study as in other studies.^4,16^

The difference of the knee moments during midstance between the prosthetic side and the CG in this study was 0.46 Nm/kg with a difference in gait velocity of 0.17 m/s. In general walking speed influences the magnitude of joint moment peaks. However, Lelas et al.^32^ identified a parameter regression equation for this parameter. The result of this equation is that a reduction of velocity of 0.17 m/s will reduce the knee moment by 0.06 NM/kg.^32^ The difference of 0.46 Nm/kg in this study with TTs are more pronounced and therefore the reduction of the knee moment during midstance was attributed to the amputation.

The absence of a forceful push-off in prosthetic feet during late stance caused by the lack of plantar flexor muscles could explain the reduction of moments in late stance. However, Esposito et al. reported no differences for this parameter between a powered (BiOM) and a passive prosthetic foot.^14^ For the cohort analysed here, the mean stance phase knee flexion of 11.4° is was slightly more pronounced than reported in other studies with 7° to 9.5°.^4,5,16^ Generally, knee flexion in early stance is significantly determined by the prosthetic alignment and the foot properties.^26,33^ The foot designs used here varied, but in all cases the foot was the “everyday foot” of the patients. The alignment used in this study was consistently biomechanically optimised^24^ for the patient group investigated. The comparably enhanced knee flexion during stance phase and the high percentage of amputees (87%) flexing the knee were achieved by an alignment which consistently followed Blumentritt’s recommendations.^24^ This is an indicator for the importance of biomechanically optimised alignment and individually customised foot properties.

The kinematic changes compared to controls were only obvious during stance, when the prosthetic alignment and foot design are of importance. During swing the residual knee of the prosthetic limb showed no effects. This is in agreement with Sanderson et al.^7^ and Powers et al.^4^ but in contrast to the reduced peak knee flexion shown by Breakey^5^ and Rabago et al.^16^

The most inconsistent results in the literature were found with respect to kinetic compensatory adaptations on the sound side,^17^ especially the parameters that were assumed to be indicators for the risk of developing osteoarthritis.

The present study shows no difference of the external knee adduction moment on the sound side compared to non-amputees. This concurs with Lloyd et al.^12^, Rabago et al.^16^ and Karimi et al.^17^ In contrast, Grabowski et al.^13^ and Royer et al.^18^ showed an increase by trend, whereas Esposito et al.^14^ and Pruziner et al.^15^ showed a decrease by trend. None of the results shown by these studies are statistically significant. The sample of 53 subjects allows to claim equivalence within an effect size of r=0.03. Therefore, it can be generally assumed that the knee adduction moment on the sound side does not differ between TTs and non-amputees.

The findings regarding peak external knee flexion moments on the sound side were also controversial. Esposito (n=10)^14^ and Nolan (n=4)^8^ reported a significant increase, whereas Rabago et al. (n=16) showed a decrease by trend.^16^ This trend was supported with the results of this study showing a significant reduction in comparison to the controls with a medium effect. From these data with a large sample size it can be concluded that there is definitively no increase of knee flexion moment of TTs compared with non-amputees.

The vertical component of the ground reaction forces were also not consistently reported. Some studies reported that the first ground reaction force peak was significantly greater on the sound side compared to healthy controls.^10,13^ Other studies reported a statistically non-significant increase.^14,15,17^ The results of this study concur with Sanderson et al.^7^ showing no difference between the sound side of the amputees and the CG. The effect size of r=0.04 also supports the null hypothesis that the two samples come from the same population and therefore show no differences.

An essential factor influencing the sound side lower limb joint loading of TT amputees is the prosthetic alignment.^24^ In this context, Grumillier demonstrated the influence of systematic prosthetic mal-alignment. Particularly, the sound side’s hip work was increased, when the prosthetic foot was internally rotated.^23^ Pinzur showed higher forces and moments on the sound side, when tilting the socket from an optimally aligned prosthesis.^34^

With a biomechanically optimised alignment as defined by Blumentritt^24^ in the present study and in the results of Karimi et al.^17^ no significant increase of knee moments and ground reaction forces could be measured on the sound side. Furthermore, Karimi et al. showed no significant increase of joint contact forces calculated by a musculoskeletal model in the intact knee joint of TTs. Hence, they could neither find any “biomechanical indicator for a possible early onset of osteoarthritis”.^17^ Therefore, it seems reasonable to assume that an appropriately aligned prosthesis does not cause overloading of the sound side during walking. This concurs with Hurley et al., who analysed the load of the contralateral limb in TT gait.^35^

It is questionable whether other parameters could explain the higher risk of knee osteoarthritis on the sound side knee of TTs. Proebsting et al. discussed the influence of sound side knee load during other activities e.g. hopping or walking with crutches without prosthesis.^21^ Although an influence of trauma, infection or rheumatism on knee osteoarthritis is generally known.

### Limitations

It should be noted that the amputees in the analysed group used different models of prosthetic feet. Furthermore, the amputees’ K-levels varied (Table 2). However, since the aim of the study was to evaluate the general gait of a group of TTs, we did not want to limit ourselves to investigating the specific gait with only one foot model or in one selected K-Level and therefore decided to use a heterogeneous patient group.

## CONCLUSION

The biomechanical data measured for the prosthetic side in a cohort of 53 unilateral TT amputees concur with findings of other studies. These results indicate that besides the missing plantarflexion of the foot at late stance, the residual knee joint is generally affected with respect to a decreased sagittal plane moment and reduced knee flexion during stance phase.

The parameters influencing the risk of developing knee osteoarthritis are discussed controversially in the literature for the sound side, but are by contrast not increased in the cohort of 53 unilateral TT amputees investigated here. Hence, we deem it reasonable to assume that an appropriate prosthesis will reduce the likelihood of overloading the knee on the sound side during normal walking. Nevertheless, other influencing factors next to biomechanical parameters during level walking (e.g. trauma, infection, rheumatism, etc.) could explain the higher risk of developing knee osteoarthritis in the sound side knee of TTs.

## DECLARATION OF CONFLICTING INTERESTS

Eva Pröbsting, Malte Bellmann, Thomas Schmalz and Andreas Hahn are employees of Ottobock, the manufacturer of prosthetic components. The authors alone are responsible for the content and writing of the paper.

## ETHICAL APPROVAL

The study is in full accordance with the requirements of the German medical device act and data protection legislation.

## AUTHOR CONTRIBUTION

- **Dipl.-Ing (FH) Eva Pröbsting**,
  investigated, analysed the data and wrote the manuscript
- **Dr. Malte Bellmann**,
  investigated, analysed the data and reviewed
- **Dr. Andreas Hahn**,
  conceived the idea of the work and reviewed
- **Dr. Thomas Schmalz**,
  investigated, analysed the data and reviewed
